# Automatic search for topological materials shifts paradigm

**DOI:** 10.1093/nsr/nwz024

**Published:** 2019-03-12

**Authors:** Chen Fang

**Affiliations:** 1 Beijing National Laboratory for Condensed Matter Physics and Institute of Physics, Chinese Academy of Sciences, China; 2 Songshan Lake Materials Laboratory, China; 3 Kavli Institute for Theoretical Sciences, Chinese Academy of Sciences, China; 4 CAS Centre for Excellence in Topological Quantum Computation, China; 5 Physical Science Laboratory, Huairou National Comprehensive Science Center, China

Topological materials are known for their exotic properties. Topological insulators have gapless modes on their surfaces despite having perfectly insulating bulk, and these surface modes cannot be gapped or localized as long as time-reversal symmetry is preserved [1]; topological semimetals host ‘Fermi arcs’ on their surfaces and exhibit quantum anomalies in their bulk [2]. The physical quantity underlying these distinct properties is the topological invariant, the defining quantum number of any topological material. In an electronic material such as semiconductor or semimetal, the topological invariant is a global quantum number determined by the wave functions of all constituent electrons as a whole, and its value is stable against any symmetry-preserving perturbation. The complete set of topological invariants, usually several integers or integers modulo *n*, for a given material determine all its topological properties. Therefore, successful numerical prediction of new topological materials critically depends on the evaluation of the topological invariants.

The evaluation is, however, technically challenging: the types and forms vary with different combinations of symmetries including time reversal, charge conservation and any one of the 230 space groups of lattice symmetry, and some invariants take highly involved forms. This difficulty has hindered a comprehensive search for topological materials in large materials databases.

In 2017, two independent works established that general relations exist between the band topology and the irreducible representations of symmetry groups at high-symmetry momenta in the Brillouin zone [3,4]. In particular, given the combination of irreducible representations of all valence bands in any non-magnetic material, the new theories show whether the material has non-trivial band topology. This qualitative diagnosis has been further developed into a quantitative one in more recent works [5–7], by which we not only know if non-trivial topology is present but also (i) whether it belongs to a topological insulator or a topological semimetal and (ii) the possible values of all topological invariants of the insulator or the minimal configurations of topological nodes of the semimetal, where ‘minimal’ means that they have the smallest number of nodes. It should be pointed out, however, that not all topological data can be inferred from symmetry representations, and there are cases where additional calculation is needed to determine the invariants. Since the diagnosis only depends on the symmetry representations at high-symmetry-crystal momenta, it cannot detect non-trivial topology induced by band inversions at generic-crystal momenta. For example, for a space group having both fourfold rotation axis and inversion, the diagnosis determines the mirror Chern numbers up to a multiple of four. For another example, the Weyl semimetal TaAs would be diagnosed trivial because all band inversions happen at generic-crystal momenta.

**Figure 1.**
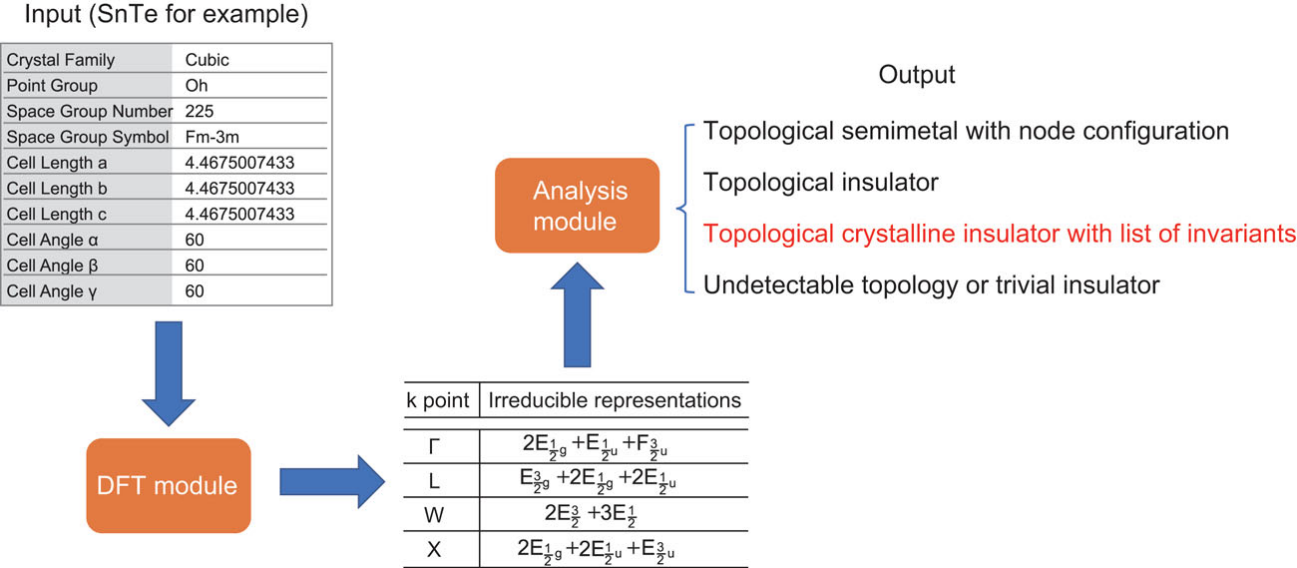
A simplified streamline description of the diagnosis method according to [11–13]. The crystal structure and atomic positions of a non-magnetic crystal (SnTe in this example) are fed into the density-functional-theory module for first-principles calculation (VASP in this example). The latter yields the irreducible representation decomposition of all valence bands at high-symmetry-crystal momenta. These symmetry data are then fed into the analysis module, which maps the symmetry data to topology data according to [3–7]. The topological data include a label (topological semimetal, topological insulator, topological crystalline insulator) and the minimal configuration of topological nodes (if topological semimetal) or a list of topological invariants (if topological crystalline insulator).

The quantitative mapping between symmetry and topology enables an automated diagnosis scheme for any non-magnetic topological material on the first-principles level (see Fig. 1 for a schematic of the process). The modern tools for band structure calculation, such as the Vienna *ab initio* Simulation Package or WIEN2k, with some modification, can automatically find the irreducible representation to which a band belongs to at any momentum in the Brillouin zone. Feeding this symmetry information into the mapping, one first determines if any band touching or crossing exists at high-symmetry points or high-symmetry lines by checking the symmetry representations of the valence bands against the compatibility relations [3], the necessary conditions for the bands to be gapped along high-symmetry lines. If there is no band touching or crossing, the symmetry information can be converted into a set of symmetry indicators [4], an intermediate result that may be considered as ‘lossless compression’ so far as band topology is concerned. The indicators are mapped to the topological invariants [5,6], from which we know if the material is a topological insulator, a topological semimetal having band crossing(s) away from high-symmetry lines [7], or a trivial insulator.

The new scheme of diagnosis is applied to finding a particular topological state (3D topological crystalline insulators with 1D edge states, or ‘high-order topological insulators’) among all materials having space group 139 [8], identifying several candidates in the Ca_2_As materials family. The scheme is also shown to be useful in finding new topological crystalline insulators and topological semimetals in eight [9] and five space groups [10], respectively. In July 2018, three research teams independently completed a sweeping search for topological materials in large materials databases such as the Inorganic Crystal Database and the Materials Project. Zhang *et al.* [11] scanned 29 171 non-magnetic crystals and found 8056 topologically non-trivial, classified into 5005 topological semimetals and 3051 topological (crystalline) insulators together with their respective topological invariants; Vergniory *et al.* [12] ran the diagnosis through all 26 938 high-quality crystals registered on the Inorganic Crystal Database, and theoretically discovered 2689 topological (crystalline) insulators and 3010 topological semimetals; and Tang *et al.* [13] chose 17 528 materials from the same database for calculation, and listed 423 topological (crystalline) insulators and 489 topological semimetals as ‘nearly ideal’ candidates. The main method of the three teams is the same, while the implementations vary in a few aspects: Zhang *et al.* include the topological diagnosis both with and without spin–orbit coupling, the latter relevant for light-atom materials; Vergniory *et al.* factor in the difficulty in crystal growth into their results; and Tang *et al.* cross-check the results using the modified Becke–Johnson (mBJ) potential calculations, appropriate for narrow gap materials.

The appearance of these ‘catalogues’ with large numbers of topological materials overturns the opinion shared by many that non-trivial band topology should be scarce in nature. It also ends the era in which scientists hunted for new candidates in the vast materials sea with their intuition and specialized expertise in topological physics. The new results may bring together various fields of condensed matter research: some of these materials have been studied for other reasons such as superconductivity, thermoelectricity or multiferroicity; hence the fact that they are also topological gives a new angle to these research areas where the interplay between topology and these desired properties can be studied.

The next goal, after the theoretical prediction, is the experimental discovery and study of new topological states. To achieve this scientists have to select from the large number of candidates that best align with their own interest, and here is where future development diverges: some are looking for more ‘ideal’ topological materials where the tops or bottoms of topologically trivial bands are away from the Fermi energy; some desire the exact opposite because trivial pockets at Fermi energy may undergo a superconducting transition and hence induce topological superconductivity; some take the challenge of studying what happens to band topology when strong electron correlation comes into play; and some focus on quasi-2D topological materials suitable for making devices. The diagnosis and the numerical predictions only apply to the non-magnetic materials; therefore, another rich yet poorly explored area of research is the non-trivial topology in magnetic materials that are protected by 1651 magnetic groups in three dimensions, the topological invariants of which have not been fully established. The opportunities are infinite.
